# New-onset Atrial Fibrillation in Patients Presenting with Acute Myocardial Infarction

**DOI:** 10.7759/cureus.4483

**Published:** 2019-04-16

**Authors:** Zafar Iqbal, Muhammad Naeem Mengal, Abida Badini, Musa Karim

**Affiliations:** 1 Interventional Cardiology, National Institute of Cardiovascular Diseases, Karachi, PAK; 2 Cardiology, National Institute of Cardiovascular Diseases, Karachi, PAK; 3 Family Medicine, Aga Khan University, Karachi, PAK; 4 Statistics, National Institute of Cardiovascular Diseases, Karachi, PAK

**Keywords:** atrial fibrillation, atrial flutter, myocardial infarction, new-onset

## Abstract

Background

Atrial fibrillation (AF) can be seen secondary to the complications after acute myocardial infarction (AMI), but it has a poor prognosis when occurs independently. These patients are prone to an increased risk of all in-hospital major cardiac complications and also at an increased risk of mortality. Therefore, it is important to quantify the burden of this aggravating complication in an otherwise lethal manifestation of acute coronary syndrome. The aim of this study was to find the frequency of AF in patients presenting with AMI and the factors associated with it.

Methods

We conducted this observational study on 216 patients who presented with AMI at the largest cardiac center of Karachi, Pakistan from July 2014 to January 2015 with AMI without a past history of AF. Patients underwent routine clinical assessment and laboratory investigations. Atrial fibrillation, detected on electrocardiographic assessment at the time of admission or during hospital stay without a prior history of persistent atrial flutter or paroxysmal atrial fibrillation, was classified as new-onset atrial fibrillation (AF).

Results

We selected a total of 216 patients, 117 (54.2%) male and mean age of 50.76 ± 6.00 years. Diabetes was found in 140 (64.8%), 164 (75.9%) were hypertensive, and 61 (28.2%) patients were smokers. ST-segment elevation myocardial infarction (STEMI) was diagnosed in 97 (44.9%) patients. The new onset of atrial fibrillation was found in 27 (12.5%) of the patients with AMI. Univariate analysis revealed a statistically significant association of new-onset AF with hypertension.

Conclusion

The new onset of atrial fibrillation was found in 12.5% of the patients presented with acute myocardial infarction. It is a significant complication in term of its frequency in AMI and it is more common in hypertensive patients.

## Introduction

Atrial fibrillation (AF) is an electrical complication, commonly observed in acute myocardial infarction (AMI) patients, with an incidence ranging from 6% to 19% [1]. However, most of these patients had pre-existing AF, the actual incidence of new-onset atrial fibrillation is as high as 5% [2-3]. Various studies have confirmed its association with increased immediate-, long-, and short-term mortality and adverse events such as heart failure (HF) and cerebrovascular accident (CVA) [1,4-6]. Pathogenesis of new-onset AF in patients with AMI is acute left atrial dilatation due to increased atrial pressure caused by an underlying factor [7]. It can be seen secondary to the complications after AMI, but it has a poor prognosis when occurs independently [8].

In a clinical setting, early detection of the AF and introduction of protective medication and therapies might greatly help to contain the progression of arrhythmia from an easily manageable condition to a completely refractory condition [7]. However, with the ‘silent’ nature of arrhythmia, for most of the times, its early detection is difficult and a significant number of patients, around one third, did not even notice the so-called asymptomatic atrial fibrillation [7].

Clinically, AF is generally associated with various risk factors such as advanced age, HF, hypertension, valvular heart disease, obesity, cardiomyopathies and thyroid dysfunction [9-10]. AF leads to various clinically adverse conditions such as mortality, cerebrovascular accident, and other thromboembolic events including HF, left ventricular (LV) dysfunction, reduced exercise capacity and quality of life [11]. The risk of AF increases with an increase in age; a prevalence of 0.5% was reported at 40 to 50 years of age and 5% to 15% at 80 years of age [7].

Clinical management is challenging, for the cases with AMI complicated by AF, due to the lack of guidelines and data regarding embolic risks and bleeding and suitable antithrombotic treatment [12-13]. In a majority of the cases, AF is chronic in nature, while new-onset AF develops occasionally. There are substantial differences in the pathophysiological mechanisms, management, and clinical impact of both conditions [14-17]. A very limited data on new-onset AF in AMI is available for the Pakistani population. Therefore, it is important to quantify the burden of this aggravating complication in an otherwise lethal manifestation of acute coronary syndrome. The aim of this study was to find the frequency of AF in patients presenting with AMI and the factors associated with it.

## Materials and methods

This observational study was conducted at the largest cardiac center in Karachi, Pakistan from July 2014 to January 2015. We included 216 male and female patients between the ages of 20-60 years with a diagnosis of AMI, within four days of admission to the hospital. Exclusion criteria for the study were patients with a prior diagnosis of AF and patients having AF due to any other causes apart from AMI. Institutional ethical review committee (ERC) approval was taken (ERC-39/2014) and informed consent was obtained by the researcher from all the patients. Assessment of AMI was made on the basis of history, ECG, and cardiac isoenzyme (troponin-I). AMI was classified as non-ST-segment elevation myocardial infarction (NSTEMI) and ST-segment elevation myocardial infarction (STEMI). Patient demographics (gender and age) and clinical history regarding hypertension, diabetes, and smoking were obtained. Treatment and management of the diseases were planned as per the guidelines and opted management strategy, medical treatment, percutaneous coronary intervention (PCI), or streptokinase, were recorded. 

All the patients underwent continuous electrocardiography monitoring for detection of new-onset AF. Electrocardiographic criteria for the presence of AF were P-waves absence, coarse or fine fibrillatory waves, and irregularity of the RR intervals. AF, detected on electrocardiographic assessment at the time of admission or during hospital stay without a prior history of persistent atrial flutter or paroxysmal atrial fibrillation, was classified as new-onset AF. As per the AHA 2001 guidelines, an episode of AF with a minimal time of more than 30s was counted [10]. The final outcome was measured on the fourth day of admission. Frequencies and percentages were calculated for gender, diabetes mellitus, hypertension, smoking status, type of AMI, type of emergency treatment i.e. streptokinase (SK) and primary PCI, and new-onset AF. The mean and standard deviation were calculated for continuous variables such as age. Stratification was done on age, gender, comorbid including diabetes mellitus, hypertension, smoking status, type of AMI, type of treatment (medical management, SK, primary PCI). Chi-square test was applied, and statistical significance criteria were *p*-value ≤ 0.05.

## Results

We selected a total of 216 patients. There were 117 (54.2%) male and 99 (45.8%) female patients. The mean age was 50.76 ± 6.00 years. Diabetes was found in 140 (64.8%), 164 (75.9%) patients were with hypertension, and 61 (28.2%) patients were smokers. Regarding diagnosis, 97 (44.9%) patients presented with STEMI and 119 (55.1%) got admitted with NTEMI. The demographics and clinical characteristics of patients are presented in Table 1.

**Table 1:** Demographic and clinical characteristics of the patients NSTEMI, non ST-segment elevation myocardial infarction; STEMI, ST-segment elevation myocardial infarction, PCI, percutaneous coronary intervention

	Frequency [n]	Percentage [%]
Gender		
Male	117	54.2%
Female	99	45.8%
Age Groups		
36 – 45 years	46	21.3%
46 – 55 years	115	53.2%
More than 55 years	55	25.5%
Comorbid		
Diabetic	140	64.8%
Hypertension	164	75.9%
Smoking	61	28.2%
Type of Acute Myocardial Infarction (AMI)		
STEMI	97	44.9%
NSTEMI	119	55.1%
Treatment Type		
Medical Treatment	131	60.6%
PCI	12	5.6%
Streptokinase	73	33.8%

According to statistical analysis, out of a total of 216 patients with AMI, new-onset AF was found in 27 (12.5%). Univariate analysis revealed a statistically significant association of new-onset AF with hypertension, while no significant association was found between age, gender, type of AMI and type of treatment given to the patient after AMI. New-onset AF by baseline characteristics of the patients is presented in Table 2.

**Table 2:** Frequency and association of new-onset of AF *Statistically significant at 0.05 level of significance; **p-values are based on the chi-square test. AF, atrial fibrillation; NSTEMI, non ST-segment elevation myocardial infarction; STEMI, ST-segment elevation myocardial infarction; PCI, percutaneous coronary intervention

	Base [N]	New-onset atrial fibrillation	p-value**
		Percentage (n)	
Gender			
Male	117	12.8% (15)	0.977
Female	99	12.1% (12)	
Age Groups			
36 – 45 years	46	13% (6)	0.918
46 – 55 years	115	13% (15)	
More than 55 years	55	10.9% (6)	
Diabetes mellitus			
Non-diabetic	76	15.8% (12)	0.281
Diabetic	140	10.7% (15)	
Hypertension status			
Non-hypertensive	52	0% (0)	0.002*
Hypertensive	164	16.5% (27)	
Smoking status			
Non-smokers	155	15.5% (24)	0.035*
Smokers	61	4.9% (3)	
Type of Acute Myocardial Infarction (AMI)			
STEMI	97	15.5% (15)	0.234
NSTEMI	119	10.1% (12)	
Treatment given			
Medical Treatment	131	11.5% (15)	0.397
PCI	12	25% (3)	
Streptokinase	73	12.3% (9)	
Total	216	12.5% (27)	

## Discussion

AMI is one of the major health problems in both developed and developing or underdeveloped countries. Occurrence of AF in acute AMI setting is not an uncommon phenomenon; various studies have reported increased immediate-, short-, or long-term mortality, adverse events and other thromboembolic events including HF, LV dysfunction, reduced quality of life, and exercise capacity in AMI patients complicated by AF [1,4-6,11].

We conducted this study on patients admitted with the diagnosis of AMI and between 20 and 60 years of age. In our study, we found that 12.5% of patients admitted with the diagnosis of AMI developed new-onset AF; this is within the range of reported frequency in previous studies [2,4-9,13-18]. It has been observed in past studies that patients with AF have more risk factors and as a result more in-hospital complications. It is important to assess all these important confounding factors while studying the association between short- and long-term outcomes and new-onset AF [19].

The important findings of our study are AF is a common arrhythmia observed in 12.5% of the patients with AMI and patients with hypertension have more chances to develop AF. In epidemiological studies, about 10% to 16% of AMI patients have been noted to develop AF during the hospitalization and an increasing trend was observed in its prevalence over the years [20]. In a study performed by Rathore et al., AF was reported in about 22% of patients during hospitalization in the elderly population of >65 years [7]. In AMI, paroxysmal AF has been reported in about 10% of the patients when thrombolytics were not in clinical practice and in about 9% of patients after the use of thrombolytics [21]. In the DIAMOND-AMI study, for the placebo group (without dofetilide), AF developed in two percent of the patients during the first year of AMI [22].

In TRACE study, the group of patients in which angiotensin-converting enzyme inhibitors (ACE-I) trandolapril was not given, the placebo subgroup, 5.3% developed new-onset atrial fibrillation [23]. Past studies have shown that the risk of stroke, left ventricular dysfunction, and death associated with new-onset AF are increased. It has been demonstrated in a number of studies that new-onset AF is an independent predictor of not only in-hospital but also short- and long-term mortality [1,4-6,8,11]. In agreement with previous studies, we found that patients with new-onset AF were with worse baseline characteristics and that hypertension was the significant predictor of AF in these patients.

The current study has several limitations; due to the observational nature of the study, we are unable to establish causality for new-onset AF, treatment, and outcomes. Second, as all the patients were on ACE-I or ARBs, this may have altered the frequency of new-onset AF. It is suggested that more prospective and interventional studies need to be conducted, with larger sample size to find the impact and association of complication of AF in the context of AMI, to possibly prevent or manage such patient more effectively.

## Conclusions

In our study, new-onset AF was found in 12.5% of patients with AMI and most of them were hypertensive and elders. The majority of patients who developed new-onset AF after AMI had HF. In the thrombolytic era, AF remained a common and important complication of AMI. It is also suggested that these patients be managed timely with close clinical monitoring, anticoagulation, and treating AF accordingly considering AMI whenever possible.
